# Bilateral Choanal Atresia with Tessier Type 3 Facial Cleft: A Rare Association

**Published:** 2012-07-01

**Authors:** Munisamy Ragavan, ArunKumar S, Balaji NS

**Affiliations:** Department of pediatric surgery, MIOT hospital Manapakkam, Chennai- 600089, India.

**Figure:**
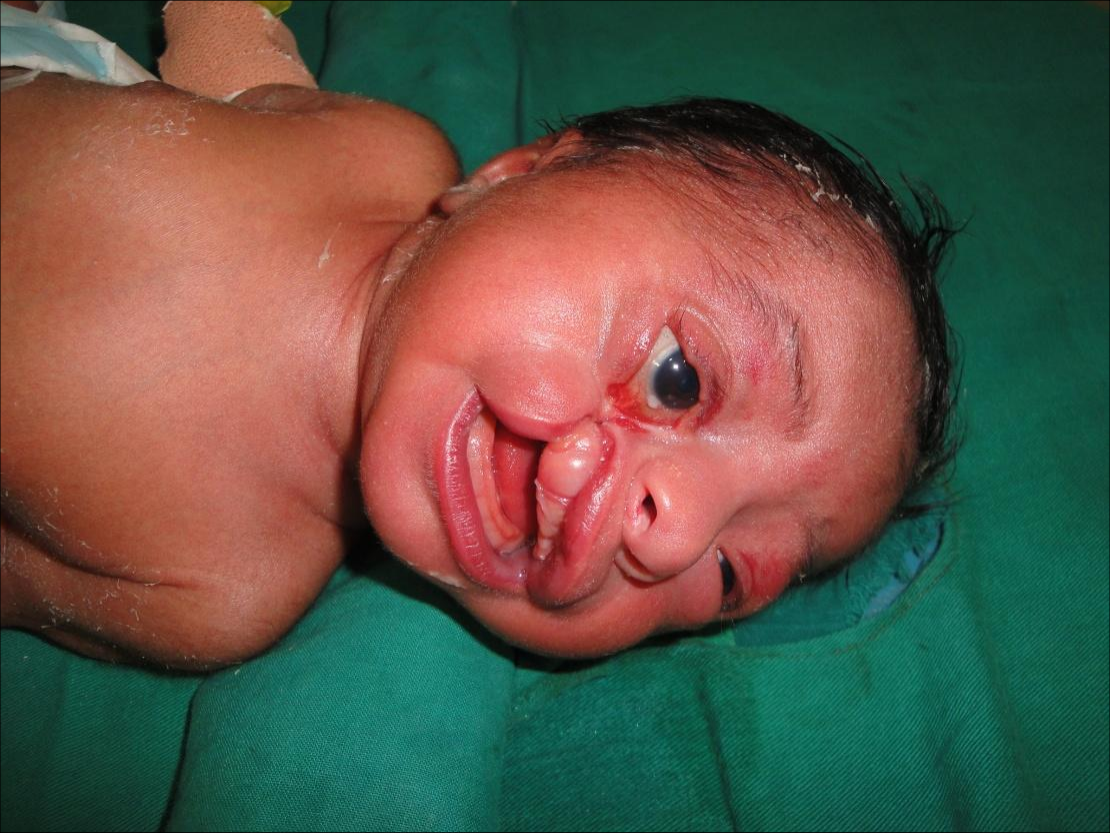
Figure 1: Baby with Tessier type 3 facial cleft and choanal atresia after resuscitation for cyanosis.

Five-day-old male neonate born to non-consanguineous parents by normal vaginal delivery was presented with deformity of lip, face and eyelid on the right side, and cyanosis. The cyanosis had improved soon after endotracheal intubation. Examination revealed deformity of upper lip, upper jaw and lower eyelid of right eye; this type of craniofacial cleft is called as Tessier type 3 (Fig. 1). There was associated bilateral choanal atresia. The baby accidently self-extubated himself after 2 days, but could maintain thereafter with oxygen administration through hood. We plan to manage choanal atresia endoscopically at least on one side first before undertaking the reconstruction of the facial cleft part.

Craniofacial cleft may involve the mouth, cheeks, eyes, ears and forehead and may continue into the hairline. In 1976, Tessier classified the clefts between 0 and 14 based on extent of the cleft using the mouth, nose and eye sockets as landmarks with the midline designated as zero [1]. The Tessier 3 and 4 facial clefts, also called naso-ocular, or nasomaxillary clefts, result from the disruption of the lateral nasal and maxillary processes [2]. Tessier cleft types 3 and 4 are most difficult and challenging malformations to correct for the reconstructive surgeon. Various techniques such as Z-plasty, local flaps, cheek rotation flap including the lower eyelid, rotation and advancement flap of the cheek, and the tissue expansion methods have been described for the repair of such clefts [3]. Sometimes, neo-conjunctivorhinostomy and cranioplasty for plagiocephaly may also be required. In the absence of any well-laid guidelines for management of such rare cases, the proposed ‘split approach' of the affected areas of the cleft into three components: a) lid component; b) lip component; and c) nasomalar component, is very helpful. Individual management of the aforesaid demarcated areas is easy as compared to the surgery of the entire craniofacial cleft [4]. A simplified classification for craniofacial clefts based on a different surgical paradigm appropriate to each regional location has been described recently [5]. Both the facial cleft and the choanal atresia seen in our case have the same embryopathogenic context- an anomaly of migration of the neural crest cells [6]. The knowledge of this association is important as the choanal atresia could influence the outcome.

## Footnotes

**Source of Support:** Nil

**Conflict of Interest:** Nil

